# Predictors of large volume paracantesis induced circulatory dysfunction in patients with massive hepatic ascites

**DOI:** 10.4103/0975-3583.70914

**Published:** 2010

**Authors:** G. Nasr, A. Hassan, S. Ahmed, A. Serwah

**Affiliations:** *Department of Cardiology, Suez Canal University, Ismailia, Egypt*; 1 *Department of Tropical and Infectious Disease Unit, Suez Canal University, Ismailia, Egypt*; 2 *Department of Medicine, Suez Canal University, Ismailia, Egypt*

**Keywords:** Circulatory dysfunction, hepatic cardiomyopathy, massive hepatic ascites

## Abstract

**Purpose:**

In patients with massive ascites, large volume paracentesis may be associated with complications as circulatory dysfunction. Selection of appropriate patients might reduce such side effects.

**Patients and Methods:**

Forty-five patients known to have liver cirrhosis and presenting with massive ascites were included. There were 27 males and 18 females, with age (mean 51.2+10.64). All patients were subjected to full history, clinical examination, complete blood picture, prothrombin time, serum albumin, total plasma protein, serum bilirubin, serum creatinine, serum electrolytes and plasma renin activity measured by radioimmunoassay. Echocardiographic evaluation for cardiac output, pulmonary artery pressure, diastolic and systolic function before and after paracentesis. Large-volume paracentesis (LVP) ranging 8–18 liters with a mean 9.9 L was performed to all patients. Paracentesis induced circulatory dysfunction (PICD) was defined as increase in plasma renin activity (PRA) of more than 50% of pretreatment value to a level greater than 7.5ng /ml/ hour on the 6th day after paracentesis.

**Results:**

The incidence of PICD in patients with massive hepatic ascites was 73.3% (87.5% with Dextran and 38.5% with albumin). There were no serious systemic or local side effects one week following LVP. Type of plasma expander and younger ages were the only independent predictors (odd ratio OR with 95% confidence interval CI, 3.01<21.79<157.58 and 0.80<.88<.97 respectively) Gender and other clinical and laboratory parameters had no influence. Neither electrolytes levels nor hematocrite value had an influence. Ascitic patients showed higher heart rate and cardiac output and lower arterial pressure that was accentuated after LVP (P < 0.01). Echocardiographic diastolic function, A wave velocity and deceleration time of the E wave were markedly increased in cirrhotic patients with tense ascites and the E/A ratio was markedly reduced (0.9 ± 0.3) but was not significantly affected by LVP. Ejection fraction had similar values of the normal patients with a tendency to increase after paracentesis. There were no changes in the left ventricular wall thickness.

**Conclusion:**

LVP is a safe and effective procedure for treatment of tense/refractory ascites. PICD is a frequently occurring silent complication following LVP. Salt free human albumin should be the plasma expander of choice especially if at least 8 liters are evacuated. Left ventricular diastolic function is altered in cirrhosis with tense ascites. This may represent an early stage of hepatic cardiomyopathy but was not affected by LVP and this was not reflected on the occurrence of PICD.

## INTRODUCTION

Ascites is the most common of the three major complications of cirrhosis together with hepatic encephalopathy and variceal hemorrhage. The development of fluid retention in the setting of cirrhosis is an important landmark in the natural history of chronic liver disease. Approximately 50% of patients with compensated cirrhosis, develop ascites during 10 years of observation[1] and 50% of them succumb in two years,[2] which has led to the inclusion of ascites as one of the indications for initiating evaluation for liver transplantation.[3]

Educations regarding dietary sodium restriction and oral diuretics have long been the mainstays of treatment of patients with cirrhosis and ascites.[4,5] This approach, has been shown to be effective in 90% of patients, however 10% lack response to high doses of diuretics or have recurrent side effects, (e.g., hepatic encephalopathy, hyponatremia, hyperkalemia, or azotemia) even when lower doses are given, and they are defined as having refractory Ascites.[6,7]

Current therapeutic strategies for refractory ascites include repeated large-volume paracentesis (LVP) with the use of plasma expanders and transjugular intrahepatic portosystemic shunts (TIPS).[8,9] Comparisons with administration of diuretics at increasing doses (maximal doses, 400mg of spironolactone per day and 160mg of furosemide per day) until loss of ascetic fluid is achieved, favors paracentesis as the method of choice,[7,10] because it is faster, more effective and is associated with fewer adverse events than diuretic therapy.[11–13]

Although TIPS is effective and prevents recurrence in patients with refractory ascites[9,14] the disadvantages of this technique which include recurrence of ascites, hepatic encephalopathy, high cost and lack of availability in some centers limit its use.[15–18] Recently the Consensus Conference of the International Ascites Club has recommended that gross ascites should be treated with therapeutic paracentesis followed by colloid volume expansion, and diuretic therapy, and that repeated LVP may be the first line treatment of refractory ascites.[19]

However, the removal of large amounts of ascitic fluid may induce an impairment of circulatory function that has been termed paracentesis-induced circulatory dysfunction (PICD), first described by Gines *et al*, 1988.[20] This is a hyperdynamic state that can appear up to 6 days after the paracentesis and is related to marked activation of the renin-angiotensin system.[21,22] It is associated with a rapid return of ascites, renal failure, and a worsened prognosis and may require medical intervention and frequent hospitalization.[23] Moreover, PICD is not spontaneously reversible and patients who develop this disorder show a reduced survival.[24]

The objectives of the present study were:

1. To estimate the rate of PICD after LVP in cirrhotic patients with tense/refractory ascites.
2. To identify the pretreatment predictors that may allow the selection of the most appropriate candidate for large volume paracentesis.
3. To asses the clinical and hemodynamic changes associated with LVP and their relation with PICD.

## PATIENTS AND METHODS

### Study design

This study was conducted as a comparative study of cirrhotic patients with massive ascites before and after large-volume paracentesis

### Patients

#### Inclusion criteria

1. Consecutive patients attending at Suez Canal University Hospital with liver cirrhosis based on clinical, laboratory data and ultrasonographic findings.
2. Tense ascites not responding to maximum dose of diuretics after at least 4 weeks of treatment.
3. Patients of both gender with an age above 20 years.
4. Consent of the patient.

#### Exclusion criteria

1. Respiratory, cardiac or renal failure
2. Noncirrhotic ascites as tuberculosis, peritoneal carcinomatosis
3. Active gastrointestinal bleeding or history in the preceding month
4. Spontaneous bacterial peritonitis and hepatic encephalopathy grade 2 or more
5. Evidence of hepatocellular carcinoma
6. Treatment with β-blockers for prophylaxis of variceal bleeding
7. Systemic sepsis within the past month
8. Platelet count less than 30×10^3^ /mm^3^
9. Prothrombin concentration less than 30%

#### Outcome factor

PICD was defined as increase in plasma renin activity (PRA) of more than 50% of pretreatment value to level greater than 7.5 ng /ml/ hour on the 6th day after paracentesis Gines *et al*, 1996[24]

#### Prognostic factors

Age, sex, severity of liver disease (Child–Pugh grade), comorbid conditions (DM, HTN, IHD), electrolytes (sodium and potassium), Creatinine level, Plasma expander given (dextran 70 or human albumin), and amount of fluid aspirated.

### Methods

Elligible patients from the outpatient clinic or emergency department were admitted to the hospital until at least one week after paracentesis. During hospital stay patients received adequate treatment according to their condition including low salt diet and diuretics.

All patients were subjected to the following:

**History and physical examination:** including vital signs, general signs of liver failure, assessment for encephalopathy, cardiac and abdominal examination.

**Laboratory investigations:** including: Complete blood picture, ALT and AST, serum albumin, serum bilirubin, PT, serum creatinine, serum electrolytes (Na and K), random blood sugar and PRA. All samples for laboratory tests were collected immediately before and at 6th days after paracentesis

The routine hematological and biochemical studies were performed using standard laboratory techniques.

Samples for PRA were obtained through a catheter inserted in a peripheral vein with the patient lying supine and rested in bed for an adequate time. The samples were collected at room temperature in tubes containing EDTA-K and immediately centrifuged. The plasma obtained was then frozen (-30°C) until analyzed. PRA was measured by a commercial kit to estimate the generation of angiotensin 1 by radioimmunoassay. The normal value for PRA was 4.5-7.5ng/ml/hour

**Abdominal ultrasonography:** was performed before the paracentesis using the HITACHI EUB 200 machine with linear probe.

**2 D-Doppler echocardiography:** Echocardiographic evaluation was performed before and on the 2nd day after paracentesis using a Hewlett-Packard phased array (Sons 1800, USA made, model: DR 53 15) ultrasonoscope using a 2.5 and 3.5 MHz phased array transducer probe using the left parasternal view (M- Mode and the apical four-chamber subcostal view (B-Mode) according to the American Society of echocardiography convention. In the apical four–chamber view a doppler recording of diastolic mitral flow was obtained by positioning the sample volume on the inflow area of the left ventricle just below the level of the mitral annulus. Measurements obtained were cardiac output, pulmonary artery pressure, wall thickness (left ventricular posterior wall thickness and inter ventricular septum thickness) left ventricular systolic, diastolic function and other cardiac abnormalities. Measurements derived from at least three cardiac cycles were averaged. The systolic function was evaluated by the ejection fraction of the left ventricle while diastolic function was evaluated by E /A ratio.

**Large volume paracentesis:** was performed on the second day of admission as described in detail by Tito *et al*, 1990.[25] An 18 – 16 g cannula with an inner sharp metal needle was inserted under strict aseptic conditions in the left lower abdominal quadrant. Once the needle entered the peritoneal cavity, the inner part was removed and ascetic fluid was drained into a sterile container. The process continued till at least 8 L - and short of total paraceentesis - were drained over a 2 hours period.

Intravenous infusion of Dextran70 6% was given during the paracentesis (100 C.C per each liter of ascitic fluid removed) or Salt-Free Human Albumin (SFHA) (8g\L per each liter of ascetic fluid removed, 50% of the dose within the first 2 hours and 50% 8 hours after paracentesis).

Fresh frozen plasma was given for patients with prolonged prothrombin time, and Platelets transfusion was given to patients with thrombocytopenia (below 40.000/c.c.)

### Statistical analysis

Descriptive statistical methods were used for data analysis and results were expressed as range or means with standard deviation (mean ± SD). Differences between groups were assessed by using parametric tests, continuous variables were assessed by using paired T test or Kruskl-Wallis test (if the variance was not homogenous) Qualitative variables were presented as frequencies, and differences were assessed by using Chi square (χ^2^)-Mc Nemur test. The level of statistical significance was set at *P* < 0.05. A best fitting logistic regression model was done to identify the independant predictors of PICD from all the significant different variables by univariate analysis.

## RESULTS

Forty five patients presenting with massive cirrhotic ascites who were consecutively admitted to the hospital were included in the study. The baseline demographic, clinical and laboratory characteristics of the study patients are presented in Table 1. They were 27 males (60%) and 18 females (40%), and their ages ranged between 26 and 79 years (mean 51.2 ± 10.64). Cirrhosis was due to chronic virus C hepatitis in the vast majority of cases and only a few had HBV or combined infection and none gave history of alcoholism. Jaundice was present in 29 (64%), peripheral edema in 37 (82%) and bleeding tendency (epistaxis / bleeding gums) in 5 (11.1%). Hypertension and Diabetes Mellitus was present in 7 (15.6%) and 9 (20%) respectively and Grade 1 encephalopathy was present in 6 (13.3%).

**Table 1:** Demographic, clinical and laboratory characteristics of patients with massive cirrhotic ascites

Demographic, clinical and laboratory variables	Value
Gender	
Male	27 (60%)
Female	18 (40%)
Age (in years)	
<40 5	(11.1)
41-50	18 (40)
> 51 22	(48.9)
Mean	51.2 ± 10.6
Jaundice	29 (64.4)
Peripheral edema	37 (82.2)
Bleeding tendency	5 (11.1)
Heart rate (bpm)	87.5 ± 8.3
Hypertension	7 (15.6)
Arterial blood pressure (mmHG)	98.4 ± 20.8
Diabetes Mellitus	9 (20)
Encephalopathy - grade <2	6 (13.3)
Hemoglobin g/dL	9.93 ± 1.83
Hematocrit	29.34 ± 5.57
Leucocytic count ×10^3^//mm^3^	6.7 ± 3.5
Platelet count ×10^3^//mm	124.0 ± 77.02
Random blood sugar mg/dL	142.89 ± 104.94
S. Bilirubin – total mg/dL	2.03 ± 1.67
S. Albumin g/dL	2.22 ± 0,46
Internation normalized ratio (INR)	1.3 ± 0.3
S.Creatinine mg/dL	1. 35 ± 0.87
S. Sodium meq/L	134.1 ± 9.4
S. Potassium meq/l	4.3 ± 0.7
Child- Pugh Class C	45 (100)

Data are expressed as No (%) and mean ± SD

The mean value of Hb (mean 9.93 ± 1.84), hamatocrit (mean 29.3 ± 5.6) and platelets count (mean 124 ± 77) were lower than normal. S albumin (mean2.03 ± 0.46) was also decreased while serum bilirubin (mean 2.033 ± 1.67) was elevated and PT (mean 14.99 ± 2.44) was prolonged. S. creatinine (mean 1.35 ± 0.87), S. Na (mean 134.1 ± 9.4) and S. K (4.3 ± 0.68) and random blood sugar (mean 142.8 ± 104.9) were within normal levels. It is worth mentioning that all the study subjects were classified as Child –Pugh class C according to the clinical and laboratory findings.

Table 2 describes the pertinent ultrasonographic findings in the study subjects. As expected most of the cases had a shrunken liver (60%) while only 24.4% had hepatomegaly. The portal vein was dilated in 55.6% of cases (mean 13.4 ± 1.4mm). The spleen was also enlarged in most cases (mean 15.5 ± 5.2cm) and the splenic vein was dilated in 60.0% of cases (mean 10.05 ± 4.3mm).

**Table 2:** Ultrasound findings of the patients with massive cirrhotic ascites

Ultrasound findings	Character
Liver:	
Normal	7 (15.5)
Enlarged	11 (24.4)
Shrunken	27 (60.0)
Dilated portal vein (mm)	25 (55.6)
Diameter	13.4 ± 1.4
Range	7 – 22
Spleen size (cm)	15.54 ± 5.2
Range	10 - 42
Dilated splenic vein	27 (60.0)
Diameter (mm)	10.05 ± 4.3
Range	4.0 – 18.0

Data are expressed as No (%) and mean ± SD

The paracentesis procedure events, PRA before and after, and the frequency of PICD in the total study subjects and outcome groups are presented in Table 3. Paracentesis was done after 24 hours of admission for each patient. The volume of fluid aspirated ranged between 8 and 18 liters (mean 9.9 ± 4.0). The plasma expander used was Dextran in 32 (71.1%) and Albumin in 13 (28.9%) of patients. The mean PRA increased from 8.3 ± 6.3 ng/l/hr before to 18.3 ± 10.3 after paracentesis and PICD occurred in 33 (73.3%) of patients.

**Table 3:** Descriptive data of paracentesis procedure and frequency of PICD for the patients

Variable	Total	PICD (n=33)	No PICD (n=12)	*P* value
Aspirated volume - Liter				
Range	8.0 - 28 9.9 ± 4.0	8.0 - 28.0 10.4 ± 4.6	8.0 -10.0 8.5 ± 0.7	0.09
Plasma expander used				
Albumin	13 (28.9)	5 (15.20)	8 (66.7)	0.002*
Dextran	32 (71.1)	28 (84.8)	4 (33.3)	
Plasma rennin activity (ng/ml/h)				
Before	8.3 ± 6.3	8.98 ± 5.22	7.27 ± 7.85	0.43
After	18.3 ± 10.3	23.97 ± 6.62	9.09 ± 8.59	<001*
PICD N (%)	33 (73.3)	33 (100)	-------	

Data are expressed as No (%) and mean ± SD PICD = paracentesis induced circulatory disfunction

* = significant

Accordingly the patients were classified into the 2 outcome groups; with and without PICD. As shown in Table 3 there was no significant difference in mean volume of aspirated fluid between the 2 groups. However, there was a highly significant statistical difference in the type of plasma expander used between them (*P* = 0.002). Out of the patients who received Dextran, 28/32 patients (87.5%) developed PICD representing 84.8% of this group. While only 4/32 (12.5%) who received it did not develop PICD representing 33.3%. of this group. On the other hand 5/13 patients (38.5%) who received albumin developed PICD and 8/15 (66.7%) did not, representing 15.2% and 66.7% of their respective groups.

Although PRA was slightly higher at baseline among the group that developed PICD, the difference was not statistically significant. The mean PRA increased from 8.98 ± 5.22 to 23.97 ± 6.62 in those who developed PICD and only from 7.27 ± 7.85 to 9.09 ± 8.59 in those who did not and the difference post paracentesis was highly statistically significant (*P* < 0.001).

Comparison of the possible risk factors of PICD between the outcome groups is shown in Table 4 and 5.

**Table 4:** Comparison of demographic and clinical data between those who developed PI CD and those who did not

Variable	PICD (n=33)	No PICD (n=12)	*P* value
Age	48.8 ± 9.6	57.8 ± 10.9	0.01*
Range	26 – 70	41 - 79	
Gender			
Females	12 (36.4)	6 (50.0)	0.50
Male	21 (63.6)	6 (50.0)	
Jaundice	6 (18.2)	4 (33.3)	0.42
Perfiral edema	26 (78.8)	11 (91.70	0.42
Hear rate (b/m) SD	89. 0 ± 8.0	92.5 ± 7.8	0.19
Hypertension	4 (12.1)	3 (25.0)	0.36
Arterial BP	98.5 ± 17.9	98.3 ± 23.7	0.98
Diabetes mellitus	4 (12.1)	5 (41.70	0.04*
Encephalopath grade <2	4 (12.1)	2 (16.7)	0.65

Data are expressed as No (%) and mean ± SD

* = Significant

**Table 5:** Comparison of laboratory findings between those who developed PICD and those who did not

Variable	PICD (n=33)	N0 PICD (n=12)	*P* value
Hemoglobin g/dL	9.8 ± 2.0	10.0 ± 1.6	0.78
Hematocrit	29.7 ±5.7	30.2 ± 5.0	0.81
Leucocytic count ×10^3^/mm	6.36 ± 3.42	7.46 ± 3.71	0.32
Platelet count ×10^3^/mm^3^	122.9 ± 66.4	127.0 ± 104.4	0.52
Random blood sugar mg/dl	123.5 ± 98.0	188.4 ± 111.9	0.03*
S bilirubin mg/dL	2.1 ± 1.7	1.9 ± 1.6	0.79
S albumin g/dL	2.2 ± 0,46	2.2 ± 0.5	0.62
Prothrombin time	14.9 ± 2.44	15.0 ± 1.9	0.34
S.creatinine mg/dL	1.3 ± 0.87	1.3 ± 0.9	0.40
S. sodium meq/L	134.1 ± 9.4	135.1 ± 9.6	0.15
S. potassium meq/L	4.3 ± 0.7	4.3 ± 0.7	0.99

Data are expressed as No (%) and mean ± SD

* = Significant

The mean age was significantly lower among those who developed PICD (48.8 ± 9.6) than those who did not (57.8 ± 10.9). There was no significant difference between the 2 groups as regards gender or clinical variables except diabetes mellitus which was significantly more frequent among those who did not develop PICD [Table 4].

Similarly, there was no significant differences between hematological, biochemical and electrolyte laboratory variables among those who developed PICD and those who did not, except for the mean random blood glucose which was significantly higher among those who did not develop PID (188.4 ± 111.9) than those who did (123.5 ± 98.0) [Table 5].

The best fitting stepwise logistic regression model for occurrence of circulatory dysfunction as a dependant variable, verses various personal and disease characteristics as independent variables [Table 6], has identified younger age and Dextran (type of plasma expander used) as the only independent significant risk factors with OR and 95% CI (0.88, 0.80 – 0.97) for the former and (21.79, 3.01 – 157.58) for the latter. The 2 variables explained 51% of the model (R-square = 0.51) which was highly statistically significant (*P* < 0.0001).

**Table 6:** Best fitting stepwise logistic regression model for occurrences of circulatory dysfunction (dependant variable) and various personal and disease characteristics as independent variables

Variable	Beta coefficient	SE	*P* value	OR with 95% CI
Age (Years)	-0.13	0.05	<0.02*	0.80<.88<.97
Plasma expander (Alb=), Dextran=1	3.08	1.01	<0.005*	3.01<21.79<157.58
Constant	5.83	2.59	<0.05*	

* staitically significant Dependant variable: Circulatory dysfunction (1=yes, 0=no) R-square: 0.51 Model Chi-Square: 19.21, *P* <0.0001 Variables entered and excluded: Diabetes, ALT, AST, Cardiac disease, Na, K, S Creatinine, mean BP, volume aspirated, US findings and haemodynamic status at base line.

The haemodynamic status of the study patients before LVP (at base line) and 48 hours after are shown in Table 7. At base line the mean heart rate (HR) (87.5 ± 8.3) and cardiac output (COP) (7.5 ± 1.9) were higher than the normal range, while mean arterial pressure (98.4 ± 20.8) was lower than normal,. The ejection fraction (EF) (58.7 ± 9.99), the posterior wall thickness (PwT0) (1.0 ± 0.2) and inter ventricular wall thickness (IVwT) (1.0 ± 0.2) were within normal limits. The EA ratio (0.9 ± 0.3) was lower than normal values and pulmonary artery pressure (PAP) (31.7 ± 15.2) was markedly increased. Forty eight hours after LVP there was significant increase in HR to (95.5 ± 9.5) and COP to (8.3 ± 2.3) and significant decrease in MAP to (90.7 ± 14.4). On the other hand there was no significant change in EF, EA ratio, PAP or PwT and IVwT.

**Table 7:** The hemodynamic changes in the study patients at baseline and 48 hrs after LVP

	Baseline		48 hrs PP	
	Range	Mean ± SD	Range	Mean ± SD
HR (bpm)*	70-110	87.5 ± 8.3	75-120	95.5 ± 9.50
MAP (mmHg)*	65 -155	98.4 ± 20.8	68 -140	90.7 ± 14.4
COP (L/M)*	4 -11.3	7.5 ± 1.9	4.5 -14.7	8.3 ± 2.3
SPR		13.12		10.93
EF %*	32 -77	58.7 ± 9.99	27 - 77	61.1 ± 11.6
EA ratio	0.5 -1.6	0.9 ± 0.3	0.5 - 1.6	0.9 ± 0.3
PAP	34 -90	31.7 ± 15.2	34 - 90	31.5 ± 13.7
PwT (cms)	0.8 -1.5	1.0 ± 0.2	0.8 – 1.5	1.0 ± 0.3
IVwT (cms)	0.6-1.6	1.0 ± 0 .2	0.6 – 1.6	1.0 ± 0 .2

* = Significant: P < 0.05

Table 8 shows comparison between those who developed PICD and those who did not as regards hemodynamic status at base line, 48 hours post-LVP and the mean before/after change. There was no statistically significant difference in haemodynamic parameters between the 2 groups before or after LVP except HR and IVwT which were significantly slightly higher among those who did not develop PICD only at base line (*P* = 0.03 and 0.04 respectively). The HR increased (8.3 ± 0.97 and 6.92 ± 1.57 respectively, *P* = 0.001 for both) and COP increased (0.9 ± 0.22, *P* =0.001 and 0.88 ± 0.37, *P* =0.38 respectively) and MAP decreased (-8.38 ± 2.6, *P* =0.002 and -6.42 ± 3.22, *P* = 0.071 respectively). There was no significant change in the other echcardiographic parameters namely: EF, E/A ratio, PAP, PwT or IVwT among the 2 groups.

**Table 8:** Comparison of hemodynamic changes between patients who did and did not develop PICD at base line and 24 hrs post-paracentesis

Variable	Baseline mean ± SD			48 hrs post paracentesis mean ± SD			Before/After difference Mean ± SEM (*P* value)	
	PICD (n=33)	N0PICD (n=12)	*P* value	PICD (n=33)	N0 PICD (n=12)	*P* value	PICD (n=33)	N0 PICD (n=12)
HR (bpm) Range	85.8 ± 8.2 70.0 - 110.0	91.8 ±6.6 82.0 - 110.0	0.03*	94.1 ± 8.9 82.0-120.0	98.8 ± 10.5 75.0-120.0	0.14	8.30 ± 0.97 (*P*=0.001)*	6.92 ± 1.57 (*P*=0.001)*
MAP (mmHg) Range	98.5 ±17.9 75.0 - 140.0	98.3 ± 23.7 65.0 - 155.0	0.98	89.6 ± 11.6 70.0-115.0	91.9 ± 18.3 68.0-140.0	0.63	-8.86 ±2.60 (<0002)*	-6.42 ±3.22 (0.071)
COP (L/MO Range	7.4 ±1.8 4.0-11.3	7.7 ± 2.2 4.5-11.1	0.67	8.3 ±2.0 4.5-11.9	8.6 ±3.1 4.7–14.7	0.73	0.90 ± 0.22 (<0001)*	0.88 ± 0.37 (0.038)*
EF Range	59.6 ± 9.0 32.0-75.0	56.1 ± 12.0 40.0-77.0	0.30	62.7 ± 10.9 27.0-74.0	57.0 ± 12.4 40.0 - 77.0	0.14	3.11 ±1.66 (0.071)	0.92 ± 1.23 (0.47)
E/A ratio Range	0.9 ±0.2 0.5-1.4	0.9 ± 0.3 0.7 - 1.6	0.93	0.9 ±0.3 0.5-1.6	0.9 ±0.3 0.7 - 1.6	0.87	-0.01 ±0.02 (0.53)	0.004 ± 0.004 (0.34)
PAP Range	32.8 ± 14.8 15.0-90.0	31.4 ± 13.3 15.0-60.0	0.78	31.1 ± 13.2 15.0 -85.0	32.4 ± 15.0 15.0-63.0	0.78	-1.68 ±0.78 (0.039)	0.93 ± 1.20 (0.45)
PwT (cms) Range	1.0 ±0.2 0.8-1.5	1.1 ±0.2 0.8 -1.4	0.29	1.0 ±0.1 0.8 -1.5	1.0 ±0.2 0.8-1.4	0.38	-0.007 ± 0.01 (0.71)	0.03 ± 0.03 (0.34)
IVwT (cms) Range	1.0 ±0.10. 0.6-1.2	1.1 ±0.2 0.9 - 1.6	0.04*	0.9 ±0.1 0.6-1.2	1.1 ±0.2 0.9-1.6	0.03	-0.1 ±0.02 (0.49)	0.02 ± 0.002 (0.63)

Data are expressed as mean ± SD

* =Significant

## DISCUSSION

Therapeutic paracentesis of 6 liters or more of ascetic fluid is a recommended option for the treatment of tense or refractory cirrhotic ascites. However, it has been linked with circulatory dysfunction associated with increased plasma rennin activity. This study was designed to assess the frequency and risk factors of parcentesis induced circulatory dysfunction (PICD) in our patients and to assess the haemodynamic and echocardigraphic changes associated with LVP and their relation with PICD. Forty five patients with post-hapatic cirrhosis with Child Pugh grade C and massive or refractory ascites were subjected to LVP. The clinical, laboratory and ultrasonographic findings of the study group are quite typical of this category of patients.

All of the patients experienced marked relief of the abdominal distension following the LVP and none developed serious complications in the form of SBP, sepsis, bleeding or increase in the grade of encephalopathy until discharge after one week at least. Procedure-associated risks have been reported to be low and include a 1% chance of significant abdominal-wall hematoma, 0.01% chance of hemoperitoneum, and a 0.01% chance of iatrogenic infection related to paracentesis,[26,27] but none of these occurred in this study. Degricija *et al*, 2003[28] also reported no systemic or local complications after removal of 6 liters of ascetic fluid in 50 patients. Several large randomized, controlled trials have shown that repeated large-volume paracentesis is safer and more effective for the treatment of tense ascites compared with larger-than-usual doses of diuretics.[29–31] Total paracentesis has also been shown to be as safe as repeated partial paracentesis and to shorten the period of hospitalization and may even be performed on an outpatient basis.[25] The fact that none of the patients who developed PICD had any complications attests that this disturbance is usually silent on the short term and so has been considered by some authors to be a “cosmetic disturbance”,[32] although it has been associated with a rapid recurrence of tense ascites and shorter survival.[24]

According to the definition 73.3% of the patients developed PICD. This figure is higher than reported by other studies using plasma expanders. It has been reported that PICD occurs in 80% of patients not receiving plasma expenders.[33] This might be attributed to the fact that over 70% of our patients received Dextran 70 as a plasma expander and only less than 30% received Salt Free Human Albumin (SFHA). PICD developed in 88% of those who received Dextran and only 38% of those who received SFHA This is in congruence with the finding of Planas *et al*, 1990,[34] Gines, *et al*, 1996[24] and Sola- Vera *et al*, 2003,[35] who found that the incidence of PICD was nearly double in patients receiving Dextran 70 6% compared with those who received SFHA (51% - 33% Vs 18% - 11%). An inverse relationship between the incidence of PICD and the halflife of the plasma expander used (days for Dextran- 70, and weeks for salt free human albumin) has been reported.[21] Although there was no significant difference in the mean volume of ascetic fluid removed between the 2 groups, the fact that our attempt to reach near total paracentesis, has led to the evacuation of 8 liters or more in all our patients with a mean of about 10 liters which might have contributed to the high incidence of PICD in this study.[35] This study had showed that albumin is more effective than saline in the prevention of PICD. Saline is a valid alternative to albumin when less than 6 L of ascitic fluid is evacuated.

By univariate analysis, only age, presence of diabetes mellitus (DM), and the level of random blood glucose were significantly different between those who developed PICD and those who did not. No other factors studied as predictors in this work whether demographic, clinical, laboratory orsonographic were found to be significantly different. Interestingly, those who developed PICD were younger and DM was less frequent among them. However, the logistic regression model in this study has shown that the only two independent risk factors of PICD are younger age and the use of Dextran as expender. In the recent study of Sola- Vera *et al*, 2003, only the type of plasma expander out of 28 variables including demographic, clinical data as well as liver and renal tests was found to have an independent predictive value for the development of PICD.[35] To the best of our knowledge no other study reported that age had an effect on PICD. The fact that younger age immerged as an independent predictor, merits further study of whether older age blunts the vasoactive response and hence protects against PICD. It has to be noted that age is a non modifiable risk factor since it is not possible to deny younger patients the benefit of LVP if they need it.

In this study, the echocardiographic findings at base line has shown that the patients already had a hyperdynamic circulatory state as indicated by the higher heart rate and cardiac output and lower mean arterial blood pressure than normal. These findings are in agreement with several studies of cirrhotic patients with and without ascites,[36–38] and has been explained by several factors including: increased sympathetic nervous activity, increased blood volume (increased preload), and the presence of arteriovenous communications.[39–41]

Forty eight hours after LVP the hyperdynamic circulatory state was accentuated as seen by the significant increase in HR and COP and significant decrease in MAP. These findings confirm earlier reports of heamodynamic changes early after paracentesis[42,43] that have been considered to have a favorable effect on alleviating some symptoms that characterize cirrhotic patients with ascites.[44,32] These early haemodynamic changes have been explained by the dynamics of paracentesis itself (short duration and higher flow rate of paracentesis),[45,46] local abdominal mechanical factors,[47] or a reflex of the stimulation of cardiac-volume receptors inhibiting sympathetic vasoconstrictive tone and renal release of rennin via an increase of cardiac volume.[42]

Our results show that the accentuation of the hyperdynamic circulation 48 hours after LVP occurred in both those who did and did not develop PICD although the extent of change of HR, COP and MAP was higher among those who developed PICD than those who did not. This is in accordance with the findings of Ruiz del Arbol *et al*. who demonstrated a significant inverse correlation between changes in systemic vascular resistance (SVR) and PRA on day 6 after ascites removal.[21] This finding strongly suggests that PICD could be caused by enhancement of peripheral arterial vasodilatation that characterize these patients.[47] Hence it has been suggested that the use of arteriolar vasoconstrictors as terilipressin[48,49] or noradrenaline,[50] could be useful in preventing this complication.

The hemodynamic changes observed in this study would support the recent hypothesis that immediately after paracentesis, an effective hypovolaemia due to accentuation of arteriolar vasodilatation occurs. The origin of this vasodilatation is probably multifactorial and includes an abrupt decrease in intra abdominal Pressure, a reflex mechanism via the increase in cardiac output and an increased release of nitric oxide, likely to be secondary to shear stress. In response to this vasodilatation, activation of the renin–angiotensin and sympathetic nervous systems takes place. Patients who are able to compensate for this vasodilatation in the first few days after paracentesis will not develop PICD and the levels of PRA will return to baseline. However, PICD will develop in those who are unable to compensate. The degree of hyporesponsiveness to vasoconstrictors could play an important role in this setting”.[32]

As regards the cardiac functional status at base line, there was evidence of left ventricular diastolic dysfunction as indicated by the significantly reduced E/A ratio, but no evidence of ventricular systolic dysfunction as evidenced by the normal EF and ventricular wall thickness (PwT and IVwT). These findings are similar to most other echocardiographic studies of cirrhotic ascetic patients that showed alteration in diastolic, but not systolic function.[42,50–53] In contrast other studies suggested both systolic and diastolic ventricular dysfunction in alcoholic,[54] and non alcoholic cirrhotic patients.[55,56] After 48 hours from LVP there was no change in the echocardiographic parameters of ventricular function from base line except a significant marginal increase in EF within the normal range. The latter finding is in agreement with that of Pozzi *et al*, 1997 who reported that the ventricular ejection fraction in cirrhotic patients with tense ascites was somewhat less than the control and non ascitic patients, with tendency to increase after large volume paracentesis. However, our findings are in contrast with their finding that removal of ascitic fluid by large volume paracentesis reduces the A wave velocity and increases the E/A ratio in patients with tense ascites.[56] Consequently the functional cardic parameters before or after LVP in this study did not reflect on the occurrence of PICD.[57] It is really confirmed that ascites is a feature of advanced liver disease related to cirrhosis so it really important to recognize.

## CONCLUSION

Large volume paracentesis has been found to be safe and effective in the treatment of cirrhotic patients with tense/refractory ascites. Salt free human albumin is the plasma expander of choice especially if at least 8 liters are evacuated. The ventricular diastolic faunction is alter in cirrhotic patients with tense ascites. This might represent an early compensated stage of cardiomyopathy, but was not affected by LVP and did not reflect on the occurrence of PICD.
